# Evolutionary engineering in *Saccharomyces cerevisiae* reveals a *TRK1*-dependent potassium influx mechanism for propionic acid tolerance

**DOI:** 10.1186/s13068-019-1427-6

**Published:** 2019-04-23

**Authors:** Xin Xu, Thomas C. Williams, Christina Divne, Isak S. Pretorius, Ian T. Paulsen

**Affiliations:** 1 Department of Molecular Sciences, Macquarie University, Sydney, NSW 2109 Australia; 2 CSIRO Synthetic Biology Future Science Platform, Canberra, ACT 2601 Australia; 3 KTH School of Engineering Sciences in Chemistry, Biotechnology and Health, KTH Royal Institute of Technology, 106 91 Stockholm, Sweden

**Keywords:** Adaptive laboratory evolution, Organic acid tolerance, Propionic acid, *TRK1*, Potassium uptake, Yeast

## Abstract

**Background:**

Propionic acid (PA), a key platform chemical produced as a by-product during petroleum refining, has been widely used as a food preservative and an important chemical intermediate in many industries. Microbial PA production through engineering yeast as a cell factory is a potentially sustainable alternative to replace petroleum refining. However, PA inhibits yeast growth at concentrations well below the titers typically required for a commercial bioprocess.

**Results:**

Adaptive laboratory evolution (ALE) with PA concentrations ranging from 15 to 45 mM enabled the isolation of yeast strains with more than threefold improved tolerance to PA. Through whole genome sequencing and CRISPR–Cas9-mediated reverse engineering, unique mutations in *TRK1*, which encodes a high-affinity potassium transporter, were revealed as the cause of increased propionic acid tolerance. Potassium supplementation growth assays showed that mutated *TRK1* alleles and extracellular potassium supplementation not only conferred tolerance to PA stress but also to multiple organic acids.

**Conclusion:**

Our study has demonstrated the use of ALE as a powerful tool to improve yeast tolerance to PA. Potassium transport and maintenance is not only critical in yeast tolerance to PA but also boosts tolerance to multiple organic acids. These results demonstrate high-affinity potassium transport as a new principle for improving organic acid tolerance in strain engineering.

**Electronic supplementary material:**

The online version of this article (10.1186/s13068-019-1427-6) contains supplementary material, which is available to authorized users.

## Background

Propionic acid (PA), a key building-block chemical, has been widely used as a food preservative and a chemical intermediate in plastics, pharmaceutical, cosmetic, paint, and herbicide industries [1–4]. According to the U.S. Department of Energy, PA is one of the top 30 candidate platform chemicals in use [5]. The global production of PA reached 400 kilotons in 2014 with USD 1.07 billion in revenue and was predicted to increase to USD 1.55 billion by 2020 [6]. However, as with most industrial chemicals, PA is currently derived from finite oil reserves in an environmentally-destructive manner. Microbial fermentation is a sustainable solution to petrochemical refining with the advantage that it is environmentally friendly, and can utilize renewable biomass as a feedstock [7]. Propionibacteria can produce PA from sugars via the Wood–Werkman cycle [8]. Different culture conditions and fermentation modes have been explored to increase the PA yield in propionibacteria [9]. However, further improving PA production in propionibacteria is challenging due to a lack of genetic engineering tools and strain characterization [10]. In addition, production is also constrained by slow growth rates, growth inhibition in acidic conditions, and high product purification costs [11]. Due to these limitations, it is of great interest to engineer platform microorganisms for the heterologous production of PA. *Escherichia coli* has been engineered with the acrylate pathway of *Clostridium propionicum* to synthesize PA but the titer was only 3.7 ± 0.2 mM [11].

In contrast to native PA producers, the yeast *Saccharomyces cerevisiae* is a robust cell factory that can grow at relatively low pH and can be easily manipulated using advanced genetic tools. Yeast has been engineered for the biotechnological production of various organic acids, such as lactic acid [12], succinic acid [13], 3-hydroxypropionic acid (3-HP) [14], and muconic acid [15]. PA has also been detected previously as a by-product in fermentation of *S. cerevisiae* [16]. Yeast is therefore a promising candidate for engineering PA production from sugars, and potentially from cellulosic biomass. However, product toxicity is a problem equal in significance to product yield optimization in microbial organic acid production. At external pH below the pKa of a weak acid, the undissociated (protonated) form of the acid can pass through the plasma membrane freely. In the near-neutral cytoplasm, it dissociates and releases the protons and counterions. The protons lead to intracellular acidification that affects internal pH homeostasis, lipid organization, and the function of cellular membranes [17–19]. In addition, the accumulation of anions is also toxic to yeast cells. To reduce stress, yeast cells increase proton export via plasma membrane and vacuolar H^+^-ATPases to maintain pH homeostasis in response to multiple organic acids [20–23]. Through transcriptomic analysis, several transcriptional regulators have been identified that mediate the response to organic acid stress in yeast. Overexpression of the Haa1p transcription factor enhanced acetic acid tolerance in yeast [24]. Multidrug resistance transporters and remodelling of the cellular envelope are also involved in weak acid detoxification [20]. For example, the ATP-binding cassette (ABC) transporters Pdr12p and Pdr5p have been proposed to be implicated in the efflux of the toxic counterions of hydrophilic and lipophilic weak acids [18, 20, 25, 26]. *SPI1*, encoding a glycosylphosphatidylinositol (GPI)-anchored cell wall protein, was identified to play a key role in yeast response to 2,4-dichlorophenoxyacetic acid [27], octanoic acid and benzoic acid [28].

In previous studies, the response of yeast to PA has been studied to investigate the resistance mechanism to organic acid food preservatives. Genome-wide screening of the yeast knock-out library identified that the transcription factor Rim101p, which is involved in maintenance of pH homeostasis and cell wall remodelling, is required for yeast resistance to PA stress [29]. Subsequently, metabolomics revealed changes in the abundance of amino acids, ATP, NAD^+^, glycerol, and trehalose in yeast exposed to PA [30]. However, despite the regulation by *RIM101*, the underlying mechanism of the PA response is yet to be fully understood, which impedes the development of yeast cell factories for PA production. Furthermore, despite these innate PA response mechanisms, yeast tolerance to PA is far below the levels required for industrial fermentation.

Adaptive laboratory evolution (ALE) is a widely used method to gain insights into the mechanisms of evolution and adaptive changes that accumulate under defined growth conditions for prolonged periods of selection [31]. ALE has, therefore, proven to be a powerful tool in the field of metabolic engineering, both for the elucidation of new design principles and the engineering of superior production strains. Applications of ALE include improving the growth rate [32] and product yield [33], adaption of strains to utilize non-native substrates or produce non-native products [34–36], and increasing the tolerance of strains towards a specific environmental stress [37, 38]. Combined with systems biology approaches, the relationship between genomic changes and the adaptive phenotype can be discovered by whole genome re-sequencing [39]. ALE has also been applied successfully in generating tolerant yeast strains to weak acids such as acetic acid [40], 3-HP [41], and lactic acid [42]. Despite the attractiveness of yeast-based PA production, no study has been performed to engineer the cell factory yeast with prolonged PA tolerance and mechanisms for improving yeast tolerance to PA have not been explored using ALE. Here, we demonstrate improved PA tolerance in yeast using ALE, and decipher the molecular mechanism of tolerance by whole genome re-sequencing and functional analysis.

## Results

### Adaptive laboratory evolution of propionic acid tolerance

**Fig. 1:**
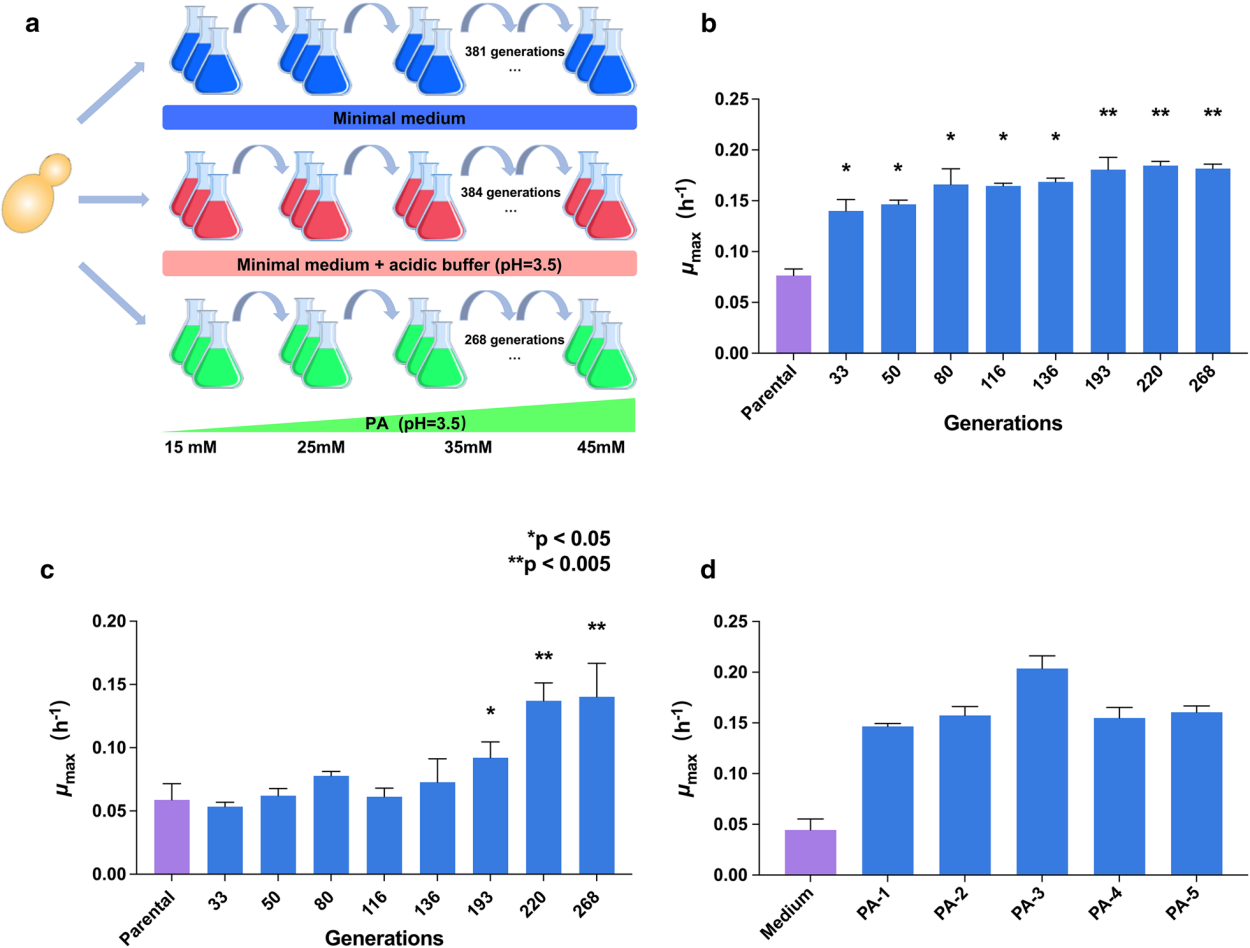
Overview of the ALE process and improved PA tolerance. Three conditions were used for the ALE experiment: minimal medium (pH 5); buffered minimal medium (pH 3.5); buffered minimal medium with increasing concentrations of PA (pH 3.5). Three independent cultures were grown under each condition (**a**). Growth rates of PA-evolved populations sampled through ALE were determined in buffered minimal medium containing 25 mM PA (**b**) and 35 mM PA (**c**). Growth rates of isolates from end-point PA-evolved populations (PA-1, PA-2, PA-3, PA-4, and PA-5) were determined in buffered minimal medium containing 35 mM PA. “Medium” represents the isolate from end-time minimal medium-evolved population (**d**). Bars and error bars represent the mean and standard deviation (SD) of triplicate cultures

Before the ALE, an initial growth test of *S. cerevisiae* CEN.PK 113-7D with PA concentrations ranging from 0 to 25 mM was conducted to identify inhibitory concentrations of PA. At 15 mM of PA, the growth rate of CEN.PK 113-7D was nearly halved, and at 25 mM of PA, the growth of the parental strain was significantly affected (Additional file 1: Fig. S1). Thus, 15 mM of PA was used as the starting concentration for ALE. Three different conditions were used for ALE in parallel: minimal medium (pH 5), buffered minimal medium (pH 3.5), and PA treated (pH 3.5). The minimal medium and buffered minimal medium acted as controls for mutations arising from genetic drift in minimal medium and from tolerance to low-pH medium, respectively (Fig. 1a). The concentration of PA was increased to 20 mM, 25 mM, 35 mM, 40 mM and 45 mM during ALE (Fig. 1a). Finally, at 45 mM, no further growth improvement was observed, and the experiment was stopped after 64 days. Fluctuations in cell density during the evolution were recorded (Additional file 1: Fig. S2) using optical density at 600 nm (OD_600nm_). The CEN.PK 113-7D strain was cultured for approximately 381 generations in minimal medium (pH 5), for 384 generations in buffered minimal medium (pH 3.5), and for 268 generations in the PA-stressed conditions (pH 3.5) (Fig. 1a). After 33 generations, the PA evolved populations (*μ*_max_ ≈ 0.14 h^−1^) showed a slightly improved fitness relative to the parental strains (*μ*_max_ ≈ 0.08 h^−1^) in buffered minimal medium supplemented with 25 mM PA (Fig. 1b). After 193 generations, the maximum specific growth rates of the evolved populations treated with 25 mM and 35 mM PA were 0.18 h^−1^ and 0.09 h^−1^, respectively, which were significantly faster than the parental strain (Fig. 1b, c). At the end of the ALE experiment, the growth rate of evolved populations treated with 35 mM PA was 2.4-fold greater than the parental strain (Fig. 1c). Five strains were isolated from end-point PA-evolved populations and designated as PA-1, PA-2, PA-3, PA-4, and PA-5. The PA-evolved strains showed 3.3–4.6-fold improved tolerance relative to the strains isolated from the minimal-medium control population (pH 5) (Fig. 1d).

### Whole genome sequencing of evolved populations and isolates

**Fig. 2:**
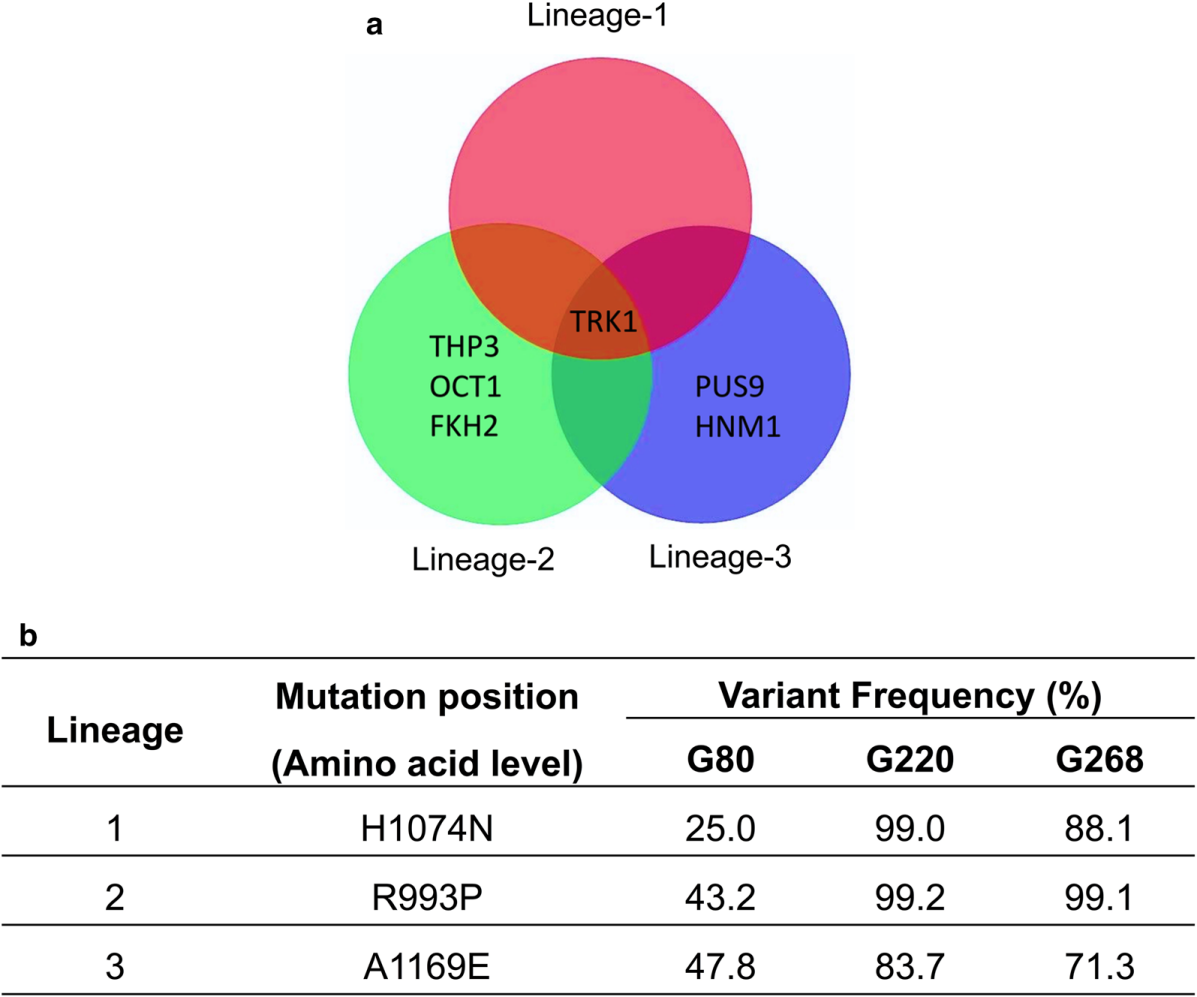
Identification of single-nucleotide polymorphism (SNPs) in PA-evolved lineages. After sequencing, Geneious Pro 9.1.3 software was used to map the reads to the CEN.PK113-7D reference genome and subsequently to perform variant calling. Non-synonymous polymorphisms in CDS regions were identified. Mutations that were also present in the parental strain, minimal medium or buffered minimal medium conditions were excluded. The Venn diagram shows that the genes with non-synonymous mutations that were identified from three end-point PA-evolved populations (**a**). The table presents variant frequencies of Trk1p in three PA-evolved lineages sampled at different generations. G represents the generation number at which the population was sequenced (**b**)

To link the PA tolerance phenotype with the genotype, whole genome re-sequencing was performed on the CEN.PK 113-7D parental strain, end-point medium control, end-point pH 3.5 buffered medium control, PA-evolved populations sampled at different generations and five PA-evolved isolates from the end-point populations. Reads from the sequenced genomes were mapped to the reference genome of CEN.PK 113-7D. Non-synonymous mutations in the parental strain and mutations in *PEX19*, *SNO4*, *KEL3* and *ALD6* that were also present in minimal medium or buffered minimal medium conditions were excluded. Non-synonymous mutations in the *TRK1* gene, encoding a high-affinity potassium transporter, were common to all PA-evolved lineages and PA-evolved isolates, suggesting that it might be the key determinant of the tolerance phenotype (Fig. 2a). Different point mutations encoding single-amino acid substitutions in *TRK1* were identified in the three independent evolved lineages: a nucleotide at position 3223 was changed from C to A (H1074N at the amino acid level) in lineage-1, a nucleotide at position 2981 was changed from G to C (R993P) in lineage-2, and a nucleotide at position 3509 was changed from C to A (A1169E) in lineage-3 (Fig. 2b). The mutations appeared after 80 generations of evolution, accounting for 25.0–47.8% of the reads from the whole evolved population (Fig. 2b). The variant frequencies became higher with increasing PA concentrations throughout the ALE experiment. At the end of the evolution experiment, the strains that contained the mutated *TRK1* alleles occupied from 71.3 to 99.1% of the whole PA-evolved populations, according to read–variant frequency (Fig. 2b). In lineage-2, non-synonymous single-nucleotide mutations were also found in *THP3, OCT1,* and *FKH2*. In lineage-3, a non-synonymous single-nucleotide mutation was also found in *PUS9*, and a premature stop-codon was identified in *HNM1*. The mutations and the functions of these genes are shown in Additional file 1: Table S3. No deletions, insertions, or duplications were observed in coding sequence (CDS) regions.

### Reverse engineering of *TRK1* mutations

**Fig. 3:**
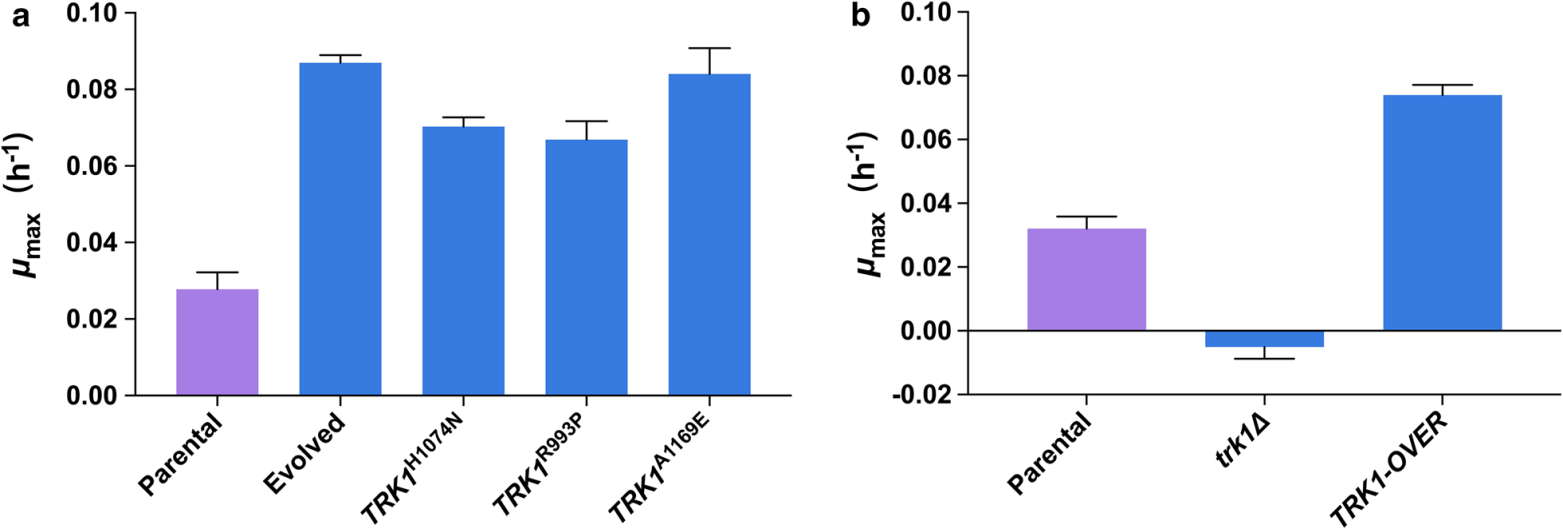
Fitness test of *TRK1* mutants treated with 35 mM PA. Three different *TRK1* point mutations were reverse engineered into the parental strain CEN.PK 113-7D to generate the *TRK1*^*H1074N*^, *TRK1*^*R993P*^, and *TRK1*^*A1169E*^ strains. Growth rates of these three strains carrying *TRK1* point mutations, the end-point PA-evolved isolate PA-1 (evolved), and the parental strain were determined in buffered minimal medium containing 35 mM PA (**a**). Growth rates of a *TRK1* deletion strain (*trk1Δ*), a *TRK1* overexpression strain (*TRK1*-*OVER*) and the parental strain were measured in buffered minimal medium containing 35 mM PA (**b**). Bars and error bars represent the mean and SD of triplicate cultures

To determine whether the single-nucleotide mutations in *TRK1* confer tolerance to PA, three different point mutations were independently reverse engineered into the parental strain to replace the original sequence. The mutations in the resulting strains, (*TRK1*^*H1074N*^, *TRK1*^*R993P*^, and *TRK1*^*A1169E*^) were confirmed using Sanger sequencing. The three reverse engineered strains showed 2.4–3-fold increased growth rates relative to the parental strain when treated with 35 mM PA at a level not significantly different to the evolved strains (Fig. 3a), fully recovering the evolved PA tolerance phenotype. To test if the *TRK1* mutations had any additive or synergistic affects, the evolved isolate PA-3 was engineered with each of the two other mutations to create the double mutant strains *TRK1*^*R993P, H1074N*^ and *TRK1*^*R993P, A1169E*^. However, no further growth-rate increase was detected under PA stress (Additional file 1: Fig. S3). Overexpression of *TRK1* was achieved by expressing the *TRK1* gene with the strong constitutive *PGK1* promoter and the *CYC1* terminator on a centromeric plasmid. The overexpression strain had an increased growth rate when treated with 35 mM PA, while the *trk1Δ* strain did not grow at all (Fig. 3b).

### Interpretation of *TRK1* mutations

**Fig. 4:**
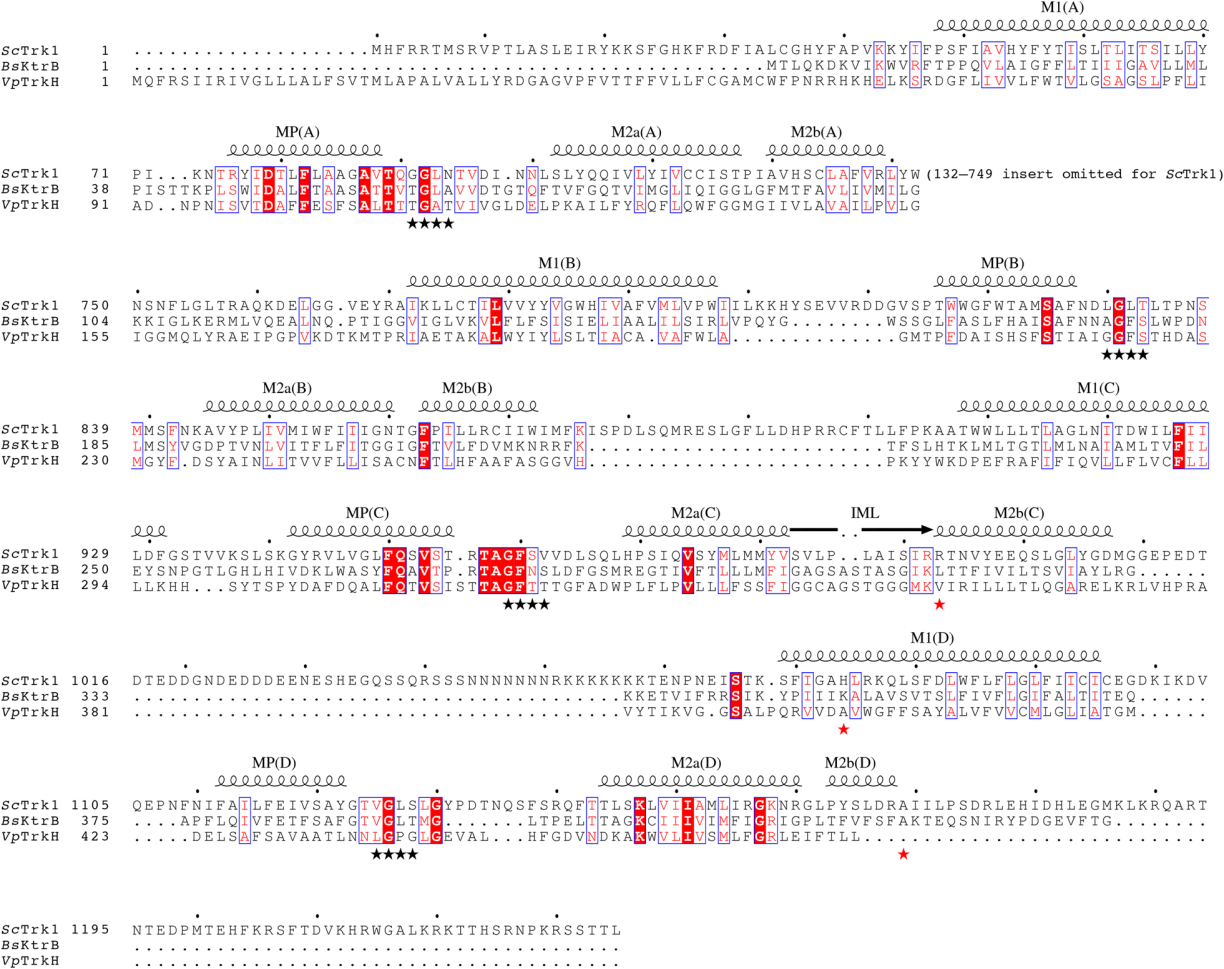
Structure alignment of *Sc*Trk1 with its homologs with known structures. The alignment was based on a structural superposition of the crystal structures of *Bs*KtrB (PDB code 4J7C [PMID: 23598340]) and *Vp*TrkH (PDB code 3PJZ [PMID: 21317882]). The selectivity filters are marked by black asterisks, the three mutations by red asterisks, and the intramembrane loop is denoted as IML. For clarity, the extensive insert (residues 132-749) in the *Sc*Trk1 sequence has been omitted. Red filled boxes indicate strict identity, and blue-framed boxes represent similarity across all aligned sequences

*TRK1*, which encodes a plasma membrane protein required for high-affinity potassium transport [43], belongs to the Trk/Ktr/HKT superfamily of ion transporters. Its structural features include four MPM motifs, where “M” denotes transmembrane helix, and “P” refers to the re-entrant pore loop preceding the selectivity filter. Each motif (Fig. 4) contains an M1 helix, a pore helix (MP), the ion-selectivity filter motif (black asterisks in Fig. 4), and helices M2a and M2b [44]. To facilitate understanding of the identified mutations, we set out to generate a homology model of *S. cerevisiae* Trk1p (*Sc*Trk1p) (Additional file 1: Fig. S4) based on a structural alignment of the two homologous transporters, i.e., *Bacillus subtilis* KtrB (*Bs*KtrB) [45] and *Vibrio parahaemolyticus* TrkH (*Vp*TrkH) [46]. The sequence identity with *Sc*Trk1p is only 22% in *Bs*KtrB and 11% in *Vp*TrkH; however, some regions are relatively well conserved, especially the helices and pore filter regions. Based on the alignment, the mutation from lineage-1 (H1074N) is positioned in helix M1 of the D motif. This position corresponds to Lys350 in *Bs*KtrB, which plays a role in securing the C-terminal tail by interactions with Lys315 and Asn119; the mutation from lineage-2 (R993P) would be at the end of the intramembrane loop (IML) and the start of helix M2b in motif C, which is likely to induce rigidity in the backbone conformation and would affect the structure and accessibility of the cytoplasmic pore. This position may also form interactions with the C-tail of another Trk1p monomer. The mutation from lineage-3 (A1169E) coincides with the first residue of the C-terminal tail. The first 15 residues of the C-tail in *Bs*KtrB ensure homodimer formation and functional association with *Bs*KtrA. Replacing the corresponding alanine in Trk1p (Ala1169) with a glutamate side chain would enable ionic interactions or hydrogen bonds that are potentially stronger than the hydrophobic interactions offered by alanine, and possibly increase oligomer stabilization [47, 48].

### Effect of potassium concentration on PA tolerance

**Fig. 5:**
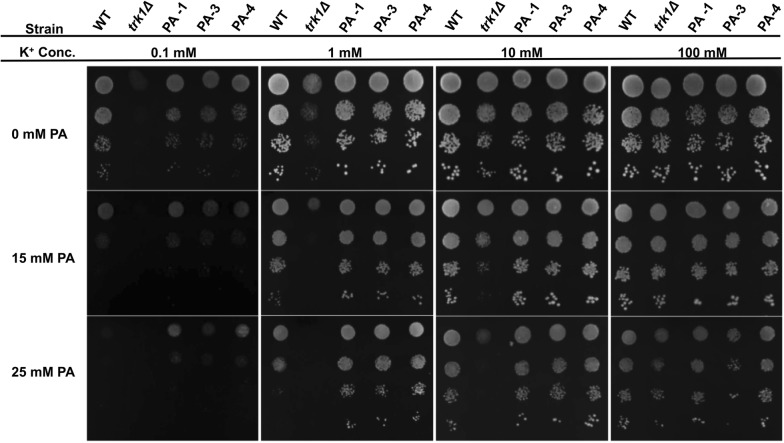
The effect of potassium concentrations and *TRK1* on the tolerance of yeast strains to PA. WT, *trk1Δ* and three end-point-evolved isolates (PA-1, PA-3, and PA-4) were grown to an OD_600_ = 0.2, serially diluted to 1:0, 1:10, 1:100, 1:1000, and then spotted on YNB agar supplemented with increasing concentrations of potassium (0.1 mM, 1 mM, 10 mM, and 100 mM) with 0 mM, 15 mM, and 25 mM PA. The plates were incubated for 3 days at 30 °C

To explore the effect of potassium concentrations and the function of *TRK1* on yeast tolerance to PA, WT, *trk1Δ* and three evolved isolates (PA-1, PA-3, and PA-4) were spotted on yeast nitrogen base (YNB) agar supplemented with increasing concentrations of potassium (0.1 mM, 1 mM, 10 mM, and 100 mM) and with 0 mM, 15 mM and 25 mM PA. On YNB agar with 0 mM PA, the three evolved strains and the WT strain had similar growth profiles at a broad potassium concentration range (from 0.1 to 100 mM) (Fig. 5). With PA added into the agar, the three evolved strains grew much faster than the WT strain, especially at 0.1 mM, 1 mM and 10 mM potassium. *Trk1Δ* generally grew slower, and it showed an obvious growth defect when PA was supplemented. Through adding increasing concentrations of potassium, growth could be restored. The growth assay was also conducted in liquid medium, which showed the same trend of improved growth with potassium supplementation under PA stress, and with *TRK1* mutation (Additional file 1: Fig. S5). These results indicate the function of *TRK1,* which encodes the high-affinity potassium transporter, is essential for the tolerance to PA in yeast, and that the evolved *TRK1* mutations enable a higher uptake of potassium under PA stress.

### The effect of potassium on tolerance to other organic acids

**Fig. 6:**
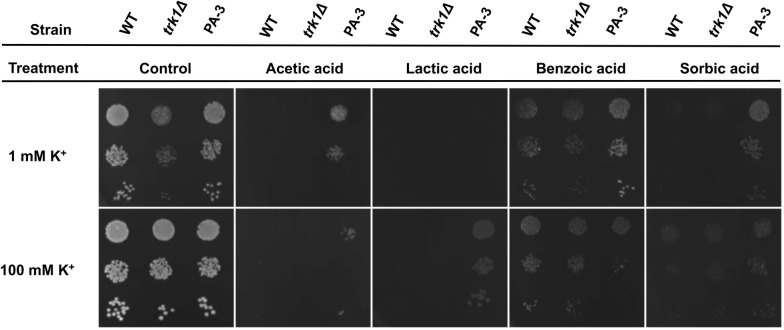
The effect of potassium concentrations and *TRK1* on the tolerance to other organic acids. WT, *trk1Δ* and the end-point-evolved isolate (PA-3) were grown to an OD_600_ = 0.2, serially diluted to 1:10, 1:100, 1:1000, and then spotted on YNB agar containing 1 mM and 100 mM potassium, without additional acids and with 83 mM acetic acid, 111 mM lactic acid, 2 mM benzoic acid, and 2 mM sorbic acid. The plates were incubated for 3 days at 30 °C

We further explored whether the tolerance mechanism can be applied to other organic acids, including the main inhibitor in lignocellulose hydrolysate, acetic acid, and three other valuable organic acids also used as food preservatives (lactic acid, benzoic acid, and sorbic acid). WT, *trk1Δ* and the evolved isolate PA-3 were tested on agar containing 1 mM and 100 mM potassium, without additional acids and with 83 mM acetic acid, 111 mM lactic acid, 2 mM benzoic acid, and 2 mM sorbic acid. In the no acid control, the evolved strain showed the same fitness as the WT at 1 mM and 100 mM potassium, while at 1 mM potassium the *trk1Δ* had a growth defect (Fig. 6). *TRK1* deletion resulted in sensitivity to all the organic acids tested. At 1 mM potassium, the evolved strain had much improved growth under the organic acid stress relative to the parental strain, especially when treated with 83 mM acetic acid, 2 mM benzoic acid, and sorbic acid. At 100 mM potassium, the evolved strain still had increased fitness relative to the WT under acetic acid and lactic acid stress, while with benzoic acid and sorbic acid, the growth of the WT and *trk1Δ* strains was recovered.

## Discussion

This study demonstrates ALE combined with whole genome re-sequencing and functional analysis can be used to unveil non-intuitive mechanisms of PA tolerance in yeast. During ALE, the concentration of PA was increased gradually to allow the accumulation of beneficial mutations as well as maintain the growth of evolved populations (Fig. 1a). The evolved strains with improved tolerance require a pre-adaption to low concentrations of PA before treatment with 35 mM PA. This could be interpreted as the cells requiring activation of the expression of inducible genes that contribute to the improved tolerance phenotype. In a previous study, *S. cerevisiae* acquired improved tolerance through laboratory evolution after continuous growth with increasing concentrations of acetic acid. However, the tolerance was rapidly lost upon storage at − 80 °C and growth without acetic acid [49]. Batch fermentation with alternating transfer to medium with and without acetic acid yielded evolved strains with constitutive tolerance. *ASG1*, *ADH3*, *SKS1* and *GIS4* were identified for the improvement of acetic acid tolerance. This on-and-off strategy allows the selection of tolerant strains that can initiate growth without pre-adaption, whereas it may also result in decreased fitness during the off phase [40].

The unique mutations in response to PA stress were obtained after excluding mutations that also existed in the parental strain, the medium-evolved condition, and low-pH medium-evolved condition (Fig. 2a). Non-synonymous mutations were found in six genes, of which *TRK1* were shared by all PA-challenged lineages and isolates (Fig. 2a). *TRK1* encodes for the high-affinity transporter for potassium uptake [43]. Three different point mutations encoding amino acid substitutions in *TRK1* were identified in the three independent evolved lineages. By reverse engineering each of the three mutations, the resulting strains fully recovered the PA tolerance phenotype, demonstrating *TRK1* is the genetic determinant (Fig. 3a). PA tolerance was not further enhanced by engineering combinations of two *TRK1* mutations in a single strain, demonstrating that the effects of the three different *TRK1* mutations on PA tolerance are not additive (Additional file 1: Fig. S3). Overexpression of the native *TRK1* gene improved the growth rate in the PA-challenged condition, showing that a higher expression level of *TRK1* also enables PA tolerance (Fig. 3b).

The other five mutated genes identified from the evolved populations only appeared in single PA-evolved lineages. A non-synonymous single-nucleotide mutation in *FKH2* and a truncation of *HNM1* were identified in evolved lineage-2 and lineage-3, respectively. Although these gene mutations were not shared among different lineages, it is noticeable that *HNM1* and *FKH2* were observed to be stress responsive in previous studies. *HNM1*, encoding a plasma membrane transporter for choline, was significantly downregulated in exposure to a multifunctional drug, pyrrolidine dithiocarbamate [50]. Fkh2, a Forkhead family transcription factor regulating the cell cycle [51], is involved in oxidative, heat [52] and acetic acid stresses [53].

A refined structural model of Trk1 and experimental validations showed that glycines within the selectivity filter are important for protein function and correct folding/membrane targeting [44]. Specifically, mutating these glycines into bulky residues leads to a growth defect in low concentrations of KCl. When highly conserved residues, such as D79 and K1147 within the M2_D_ helix and the *P*_A_ loop, were mutated to alter the charges, severe protein folding and function defects were observed as well [44]. Our sequence alignment of *Sc*Trk1p against *Bs*KtrB and *Vp*TrkH agrees well with the previous study for the more conserved regions, while there are discrepancies for other regions [44]. In our study, the point mutations H1074N, A1169E and R993P were located in helix M1, in the C-terminal tail and within or close to the intramembrane loop, respectively (Fig. 4). All appeared to play an important role in interactions associated with the C-tail, and probably increase oligomer stabilization. Given that the mutated residues are also associated with interactions that affect the pore gate, we would assume that the mutations also affect transporter function. Our results provide new insights to understand and modulate Trk1p function.

Through potassium supplementation growth assays, it was evident that insufficient potassium uptake by *trk1Δ* and the parental strain lead to susceptibility to PA stress (Fig. 5). Supplementation of higher concentrations of potassium recovered the growth and improved yeast tolerance to PA. The evolved strains showed much improved growth under PA stress, especially at low potassium concentrations. Our results suggest that the mutated *TRK1* alleles of the evolved strains improve the uptake and maintenance of intracellular potassium ions, which facilitate the detoxification of PA. When other organic acids were tested, the evolved *TRK1* mutation also conferred tolerance to acetic acid, lactic acid, benzoic acid, and sorbic acid (Fig. 6). Increasing the concentration of extracellular potassium to 100 mM enabled the growth of the WT and *trk1Δ* strains under benzoic acid and sorbic acid stresses, while it decreased the fitness of the evolved mutant slightly, indicating there is a suitable potassium concentration range for tolerance to these organic acids.

Deletion of genes related with potassium import (Trk1p, Nha1p, Arl1p) has been reported to result in susceptibility to acetic acid [54]. The food spoilage yeasts *Zygosaccharomyces bailii* and *S. cerevisiae* have been observed to accumulate potassium during long-term adaption to benzoic acid [55]. *TRK1* deletion in *S. cerevisiae* confers sensitivity to benzoic acid [55] and sorbic acid [56]. Our potassium supplementation assay is consistent with the previous studies above and also revealed the significance of potassium homeostasis in *S. cerevisiae* tolerance to lactic acid for the first time. Interestingly, although genome-wide screening of the *S. cerevisiae* knock-out mutant library demonstrated that vacuolar function and the *RIM101* pathway are important for *S. cerevisiae* resistance to PA; none of the genes identified in our study were identified previously [29]. This is not surprising given that our aims and experimental conditions were different. The previous study focused on the identification of gene deletions resulting in PA susceptibility upon short exposure to PA with the auxotrophic BY4741 strain and the Euroscarf collection of mutant strains [29]. However, our study was aimed at improving prolonged PA tolerance for future PA production in yeast, and the prototrophic CEN.PK 113-7D strain was used for ALE, which is a more robust and industrially relevant strain with thousands of genetic differences to the BY4741 strain [57].

In this study, a long-term PA tolerance mechanism was identified through ALE, and yeast tolerance to PA was demonstrated to positively correlate with the extracellular concentration of potassium and the expression level or activity of Trk1p for the first time. The tolerance mechanism identified in our study will pave the way for future engineering of PA production in yeast. *TRK1* mutations or extracellular potassium supplementation not only confers tolerance to PA but also boosts tolerance to multiple organic acids, many of which are valuable products or potent growth-inhibitors found in lignocellulose hydrolysate. In particular, acetic acid is formed from the degradation of hemicelluloses during lignocellulose pre-treatment [58]. These evolved *TRK1* mutations are, therefore, highly significant for future biorefining of organic acids and lignocellulose-based biofuel production.

One of the major weak acid toxicity mechanisms is the acidification of the cytoplasm. The export of protons relies on the activation of *PMA1*, which encodes for an H^+^-ATPase, and the H^+^ efflux is coupled with K^+^ influx. It has been reported that potassium uptake by the Trk system promotes the export of protons and alkalinizes cytosolic pH [59]. Furthermore, the undissociated form of PA impairs plasma membrane integrity, which leads to the disruption of ionic gradients and the increase of ion leakage. We hypothesized that the supplementation of higher concentrations of extracellular potassium or the mutations of *TRK1* might function in two major ways: first, they promote the export of protons, reducing the toxicity of intracellular acidification; second, they strengthened transmembrane ion gradients (proton and potassium gradients), helping to stabilize the membrane potential and improve ion homeostasis. Potassium uptake by yeast has also been linked to protein synthesis, activation of enzymes [60, 61], regulation of oxidative phosphorylation [62], DNA replication, and the cell cycle [59, 63], which all play critical roles in weak acid tolerance [55]. In addition to tolerance to weak acids, it has been observed that KCl and KOH supplementation boosted ethanol production from yeast by improving tolerance to ethanol. The tolerance was not limited to ethanol, but also observed for higher chain alcohols, which results from strengthening the opposing potassium and proton electrochemical membrane gradient [64]. Trk1p also increases tolerance to cations by preventing cation import. Heterologous expression of *TRK1* from *Zygosaccharomyces rouxii* confers high lithium tolerance in *S. cerevisiae* [65]. The functions of Trk1p have not been fully discovered and it may play an important role in tolerance to other stress conditions. These results, along with our own, demonstrate the complex role that Trk1p plays in stress tolerance in yeast, including organic acid tolerance and alcohol tolerance, with *TRK1* knock-out, overexpression, and amino acid substitutions giving different phenotypes in different stress conditions. *TRK1* and its homologs are emerging as critical components that must be considered in weak acid tolerance engineering.

## Conclusion

The results presented in this paper show that ALE can be used for the accumulation of beneficial mutations and the selection of yeast strains that are more tolerant to PA. Three convergent mutations in *TRK1* were identified using whole genome sequencing of independent laboratory evolved lineages and reverse engineering of the ancestral strain. The PA detoxification mechanism is dependent on potassium uptake and accumulation enabling maintenance of pH homeostasis and stabilization of membrane potential, and this mechanism can be applied to multiple organic acids. In the future, the toxicity associated with the bioproduction of organic acids could be reduced by engineering *TRK1,* or by increasing the potassium concentration in the fermentation medium. Conceivably, these two approaches could be employed simultaneously to further increase organic acid tolerance in a production scenario, although the use of mutated *TRK1* is preferable given that it avoids the cost of additional potassium supplementation, as well as the biological burden of *TRK1* overexpression. This work, therefore, reveals a new principle for strain engineering in the production of PA and multiple organic acids, as well as reducing the toxicity of organic acids in the fermentation of lignocellulosic hydrolysates in yeast.

## Methods

### Strains and media

Yeast strains were grown in liquid yeast peptone dextrose (YPD) broth at 30 °C, 200 rpm, streaked out on YPD agar, and incubated at 30 °C for 1 day to maintain the strains. Strains transformed with the *hphMX* marker gene were selected on YPD agar plates supplemented with 300 μg/mL hygromycin. Minimal medium containing 1× yeast nitrogen base without amino acids mix (Sigma-Aldrich Y0626), with 1% glucose (pH 5) was used for ALE. For the buffered control and PA-treated conditions, 174 mL of 0.5 M citric acid and 140 mL of 0.5 M Na_2_HPO_4_ solution were added per liter of minimal medium (pH 3.5). For potassium supplementation assays, the YNB agar was made of TRANSLUCENT K^+^-free medium (Formedium, CYN7501), with 1% of glucose, 2.4% agar and appropriate concentrations of potassium.

All the cloning experiments were conducted with *E. coli* DH5α. The *E. coli* strains were grown in Luria–Bertani (LB) broth at 37 °C with shaking at 200 rpm. *E. coli* transformants were plated on LB agar plate containing ampicillin and incubated at 37 °C overnight.

### ALE of *S.* *cerevisiae* strain CEN.PK113-7D

The haploid *S.* *cerevisiae* strain CEN.PK113-7D was used as the original parental strain for the ALE experiment. To determine the inhibitory PA concentration for evolution, an initial growth test of the parental strain in buffered minimal medium with 0–25 mM PA was conducted. For ALE, one single colony of CEN.PK 113-7D was inoculated into 5 mL YPD and grown overnight. The overnight culture was re-inoculated into 20 mL YPD at a starting OD_600_ of 0.5 and grown for another 4 h. The cells obtained from two rounds of pre-growth were used to inoculate flasks containing 10 mL of medium at an initial OD_600_ of 0.1. ALE was conducted in three different conditions, and three replicates were set in each condition. The evolution conditions were set as follows: minimal medium (pH 5); minimal medium with citric acid and Na_2_HPO_4_ buffered to pH 3.5; minimal medium with citric acid and Na_2_HPO_4_ buffered to pH 3.5 with PA at a starting concentration of 15 mM. The cultures were grown at 30 °C, 200 rpm until they reached the exponential phase before being transferred into fresh medium daily at an initial OD_600_ value of 0.1–0.2. Changes in cell density were recorded throughout the ALE. The concentration of PA was increased gradually when there was an improvement of growth and finally increased to 45 mM at the end of the evolution.

The number of generations through the evolution was estimated by adding up the ${ \log }_{2}^{{({\text{final OD}}_{ 600} /{\text{initial OD}}_{ 600} )}}$ value of each transfer. Glycerol stocks of evolved populations were made at intervals throughout the evolution. Microscopy was conducted weekly to determine whether there was any contamination in the cell culture. At the end of the evolution, single clones were isolated by serial dilution from each of the evolution lineages and plating on YNB agar.

### Fitness test under propionic acid stress

Aliquots (30 μL) of glycerol stocks of evolved populations kept at intervals were inoculated into 1 mL of minimal medium (pH 3.5) and incubated overnight with shaking before re-inoculation into 1 mL of buffered minimal medium with 15 mM PA at initial OD of 0.2 for another overnight culture. The second round of preculture was inoculated into 5 mL of buffered minimal medium with 25 mM and 35 mM of PA (pH 3.5). The experiments were repeated three times, and in each time, it was conducted in triplicates. The OD_600_ values were measured, and maximum specific growth rates were calculated.

The growth rate test of PA-evolved strains isolated at the end of the evolution was conducted with the same method above. Eight colonies were randomly picked from each of end-time PA-evolved cultures, and a total of five top performing isolates (PA-1, PA-2, PA-3, PA-4, and PA-5) were selected for further analysis.

### Whole genome sequencing and variant calling

Twenty samples submitted for whole genome sequencing were as follows: the parental strain, five PA-evolved single isolates, the intermediate PA-evolved populations sampled at two time-points, and end-time populations evolved in minimal medium, in buffered minimal medium, and in buffered minimal medium containing PA. The strains were grown overnight in YPD, and their genomic DNA was extracted with the Yeast DNA Extraction Kit (Thermo Fisher scientific, Cat No. 78870). Sequencing and library preparation were carried out by Macrogen Inc. using a True-Seq Nano kit with 470-bp inserts and paired-end Illumina HiSeq 2500 sequencing. According to the raw data report, all samples gave a quality score of Q30 above 90% and above 9 million reads and were, therefore, not trimmed. Paired end reads were analyzed using Geneious Pro 9.1.3 [66] by mapping to the CEN.PK 113-7D reference genome. This gave an average coverage of > 50×. Variant calling was performed at a minimum coverage of 10× and a minimum variant frequency of 0.05. Maximum variant *p* value was set at 10^−5^ and minimum strand-bias *p* value was set at 10^−5^ when exceeding 65% bias. Non-synonymous polymorphisms in CDS regions were identified. The mutations existing in the parental strain and in the control conditions were excluded from further analysis.

### Reverse engineering

The parental strain CEN.PK 113-7D was used for reverse engineering. The plasmids and primers used in this study are listed in Additional file 1: Tables S1 and S2. *E. coli* DH5α was used for cloning using standard techniques. Yeast DNA extraction was carried out with the LiOAc–SDS method [67] or with the genomic DNA extraction kit mentioned above. Yeast transformation was performed using the LiAc/SS carrier DNA/PEG method [68].

Re-construction of *TRK1* mutations in the CEN.PK 113-7D parental strain and the PA-evolved isolate PA-3 was conducted via CRISPR–Cas9-mediated targeting and homologous recombination. First, each of the three *TRK1* mutations was reverse engineered into the parental strain, respectively. Partial open reading frames (ORFs) of *TRK1* variants containing each of the three mutated sequences were PCR amplified with TRK1 primers. A plasmid (Cas9-gRNA-423) containing the guide RNA expression cassette, the *Streptococcus pyogenes Cas9* gene [69], and the *hphMX* marker was used for CRISPR–Cas9-mediated genome modification. The three gRNAs for targeting different *TRK1* regions were designed by annotating all potential PAM sites and guide RNAs using Geneious Pro 9.1.3 [66]. Plasmid Cas9-gRNA-pRS423 [70] containing a previous gRNA was PCR amplified with each of the three pCRISPR-TRK1-gRNA primers that have 20 nucleotides (nt) new gRNA targeting DNA sequences as 5′ extensions. The primer extensions of CRISPR-TRK1-gRNA were complementary so that the linearized PCR product could be circularized to create Cas9-TRK1-crRNA-pRS423 using Yeast Assembly [71]. The partial *TRK1* fragments and linearized PCR product of Cas9-TRK1-crRNA-pRS423 were co-transformed into CEN.PK 113-7D, respectively, and plated onto YPD agar containing 300 µg/mL Hygromycin B. Second, partial ORFs of *TRK1* variants containing each of two other *TRK1* mutations were PCR amplified with TRK1-3223 and TRK1-3509 primers, respectively. The PCR products were used to reconstruct *TRK1* mutations in the PA-evolved isolate PA-3 via CRISPR–Cas9-mediated targeting and homologous recombination as the same method above. All the site-directed mutated strains were identified and confirmed by Sanger sequencing with TRK1 seq primers.

The *trk1Δ* strain was generated by replacing the ORF region of *TRK1* with the *hphMX* cassette. The *hphMX* cassette was amplified using Hph-TRK primers from pRS426-HphMX with overhangs homologous to *TRK1*. Upstream 500-bp and downstream 500-bp overhangs of *TRK1 *were PCR amplified from gDNA with TRK1-upstream and downstream primers, which contained regions homologous to *hphMX*. The two overhangs have homologous sequences to both *TRK1* flanking regions and *hphMX* cassette, and were co-transformed with the Hph-TRK1 amplicon for deletion of *TRK1*. The *trk1Δ* mutant was confirmed by PCR with TRK1-Hph-knock-seq primers.

To enable overexpression of native *TRK1*, the ORF region of *TRK1* and the linearized pRS426 backbone were PCR amplified from yeast genomic DNA and pPGK1-CYC1t-pRS426, respectively, with overhangs to each other. The PCR products were then purified and ligated using Gibson Assembly [72]. The ligation products were transformed into *E. coli*, and the newly generated plasmids pPGK1-TRK1-CYC1t-pRS426 were extracted and characterized using TRK1-pRS426-check primers. The pPGK1-TRK1-CYC1t-HphMX cassette and linearized pRS413 backbone were amplified with corresponding primers and ligated through Gibson Assembly. After transforming into *E. coli*, the plasmids were extracted and characterized using ′pPGK1-TRK1-CYC1t-pRS413 check′ primers. Plasmid pPGK1-TRK1-CYC1t-pRS413 was transformed into the CEN.PK 113-7D parental strain for overexpression of *TRK1*.

The fitness tests of the reverse engineering strains were conducted with the same method as described above.

### Sequence alignment and structure-based homology modelling of Trk1

The coordinates for *B*sKtrB (PDB code 4J7C [45]) and *Vp*TrkH protein structures (PDB code 3PJZ [46]) were superimposed using the secondary-structure matching (SSM) algorithm in COOT [73]. The sequences for *Bs*KtrB (UniProt O32081) and *Vp*TrkH (UniProt Q87TN7) were then aligned manually to agree with the topological alignment. The *ScTrk1*p sequence (UniProt P12685) was added manually to the structure-based sequence alignment and adjusted based on sequence similarity/identity and topological features of the aligned crystal structures as well as results from consensus transmembrane-helix assignment generated by TOPCONS [74]. A starting homology model was generated automatically with the SWISS-MODEL server (https://swissmodel.expasy.org), and further rebuilt manually in COOT to agree with our manually optimized structure-based sequence alignment, and to impose good backbone and side-chain torsion angle geometry, and relieve unfavorable interactions. Figure 4 is generated by displaying the manual alignment using ESPript 3.0 (http://espript.ibcp.fr) [75].

### Potassium uptake measurement

To determine the effect of potassium concentrations and the function of *TRK1* on the tolerance to PA, the WT, *trk1Δ,* and three evolved isolates (PA-1, PA-3, and PA-4) were grown overnight in Translucent K^+^ free YNB medium supplemented with 0.5 mM KCl. The suspension was washed twice, adjusted to an OD_600_ = 0.2 and serially diluted to 1:0, 1:10, 1:100, 1:1000, then spotted with 10 μL on YNB agar (pH 4.7) supplemented with increasing concentrations of potassium (0.1 mM, 1 mM, 10 mM, and 100 mM) with 0 mM, 15 mM (pH 4.0), and 25 mM PA (pH 4.0). To investigate the effect of potassium supplementation and *TRK1* on the tolerance to other organic acids, the WT, *trk1Δ,* and the evolved isolate PA-3 were grown and diluted as the same method above, then spotted on YNB agar containing 1 mM and 100 mM potassium, without additional acids and with 83 mM acetic acid (pH 3.2), 111 mM lactic acid (pH 2.5), 2 mM benzoic acid (pH 3.7), and 2 mM sorbic acid (pH 3.0). The plates were incubated for 3 days at 30 °C. Growth rate tests of WT, *trk1Δ* and PA-3 were also performed in liquid Translucent K^+^-free YNB medium supplemented with increasing concentrations of potassium treated with 0 mM and 25 mM PA.

## Additional file

**Additional file 1: Fig. S1.** PA effect to the growth of *S. cerevisiae*. **Fig S2.** The fluctuations of yeast growth through adaptive laboratory evolution. **Fig. S3.** Fitness test of *TRK1* mutants containing different combinations of two mutations in 35 mM PA. **Fig. S4.** Cartoon showing the overall fold of the *Sc*Trk1 channel. **Fig. S5.** The effect of potassium concentrations and *TRK1* on the tolerance of yeast strains to PA in liquid culture. **Table S1.** List of plasmids used in this study. **Table S2.** List of primers used in this study. **Table S3.** Genotypic changes in the PA evolved populations.

## Abbreviations

PA: propionic acid
ALE: adaptive laboratory evolution
3-HP: 3-hydroxypropionic acid
ABC: ATP-binding cassette
GPI: glycosylphosphatidylinositol
WT: wild type
OD_600_: optical density at 600 nm
SD: standard deviation
SNPs: single-nucleotide polymorphism
CDS: coding sequence
ORF: open reading frame
*Sc*Trk1p: *Saccharomyces cerevisiae* Trk1p
*Bs*KtrB: *Bacillus subtilis* KtrB
*Vp*TrkH: *Vibrio parahaemolyticus* TrkH
IML: the intramembrane loop
MP: pore helix
SSM: secondary-structure matching
YNB: yeast nitrogen base
YPD: yeast peptone dextrose
LB: Luria–Bertani
