# Supplementary material for: Evolutionary engineering in Saccharomyces cerevisiae reveals a TRK1-dependent potassium influx mechanism for propionic acid tolerance
Source: Biotechnol Biofuels. 2019 Apr 23;12:97. doi: 10.1186/s13068-019-1427-6 (PMC6477708; doi:10.1186/s13068-019-1427-6)
Supplement: Supplementary file 1 — Additional file 1: Fig. S1. PA effect to the growth of S. cerevisiae. Fig S2. The fluctuations of yeast growth through adaptive laboratory evolution. Fig. S3. Fitness test of TRK1 mutants containing different combinations of two mutations in 35 mM PA. Fig. S4. Cartoon showing the overall fold of the ScTrk1 channel. Fig. S5. The effect of potassium concentrations and TRK1 on the tolerance of yeast strains to PA in liquid culture. Table S1. List of plasmids used in this study. Table S2. List of primers used in this study. Table S3. Genotypic changes in the PA evolved populations. [file 13068_2019_1427_MOESM1_ESM.docx]

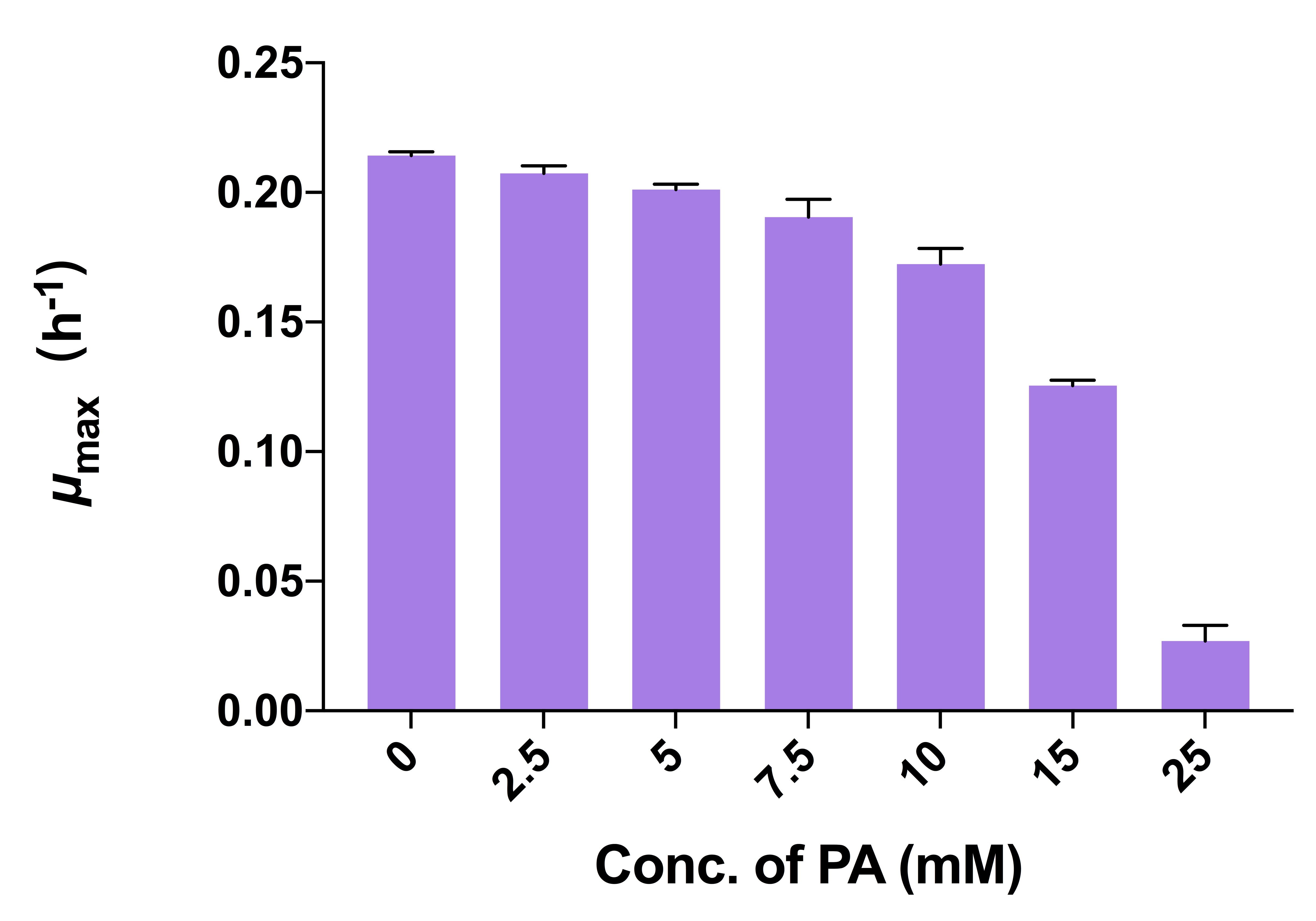


**Fig. S1. PA effect to the growth of *S. cerevisiae.*** Bars and error bars represent the mean and standard deviation (SD) of triplicate cultures.





**Fig. S2. The fluctuations of yeast growth through adaptive laboratory evolution.** Changes of cell density in minimal medium (pH 5) (**a**), in buffered minimal medium (pH 3.5) (**b**), and in buffered minimal medium (pH 3.5) with increasing concentrations of PA from 15 mM to 45 mM (**c**). Data represents the average of OD_600_ for 3 replicates and SD value is given by error bars.


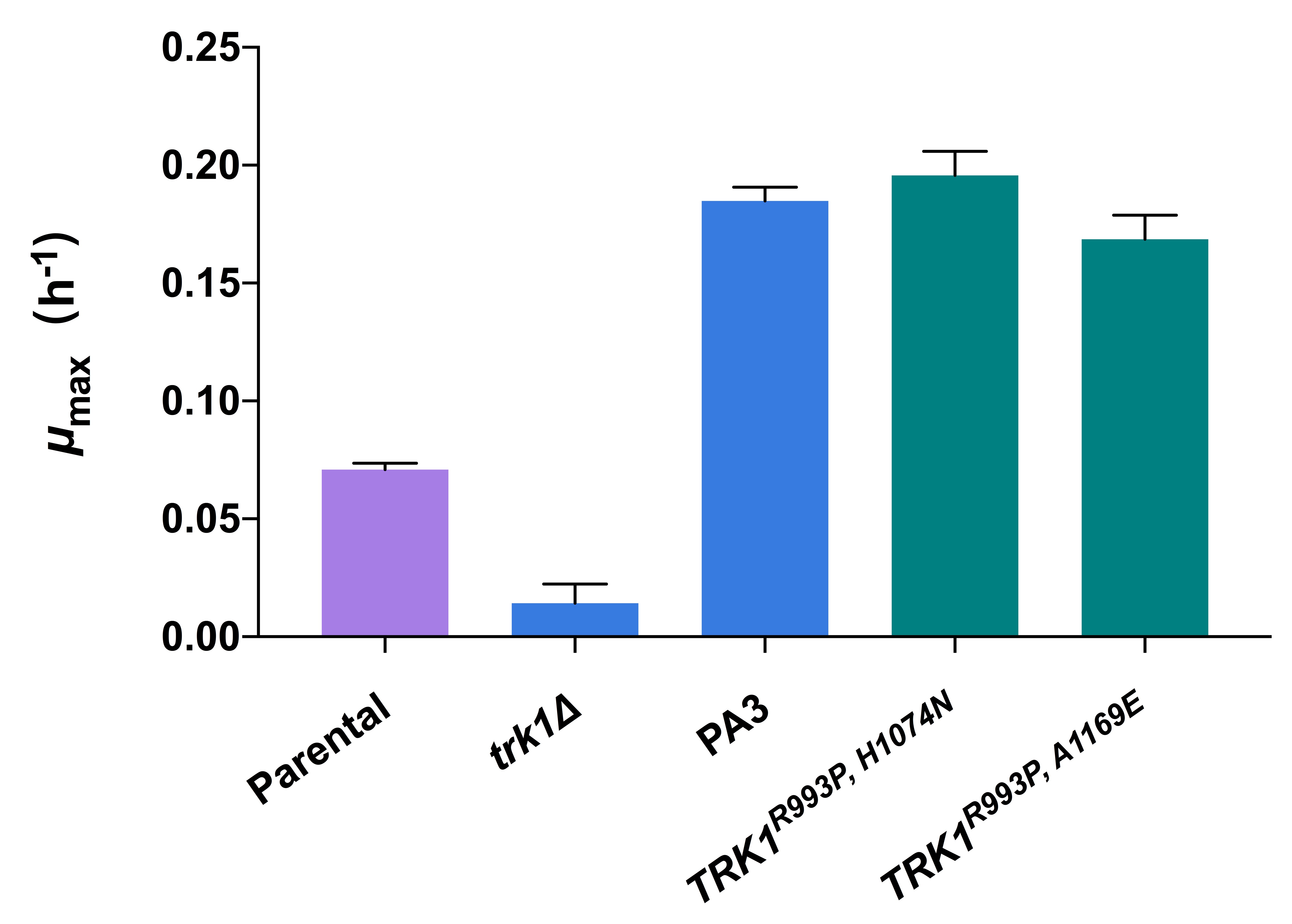


**Fig. S3. Fitness test of *TRK1* mutants containing different combinations of two mutations in 35 mM PA.** The evolved isolate PA-3 was engineered with different combinations of two *TRK1* mutations (*TRK1^R993P, H1074N^* and *TRK1^R993P, A1169E^*). Growth rates of these strains, a *TRK1* deletion strain, and the parental strain were determined in buffered minimal medium containing 35 mM PA. Bars and error bars represent the mean and SD of triplicate cultures.

**
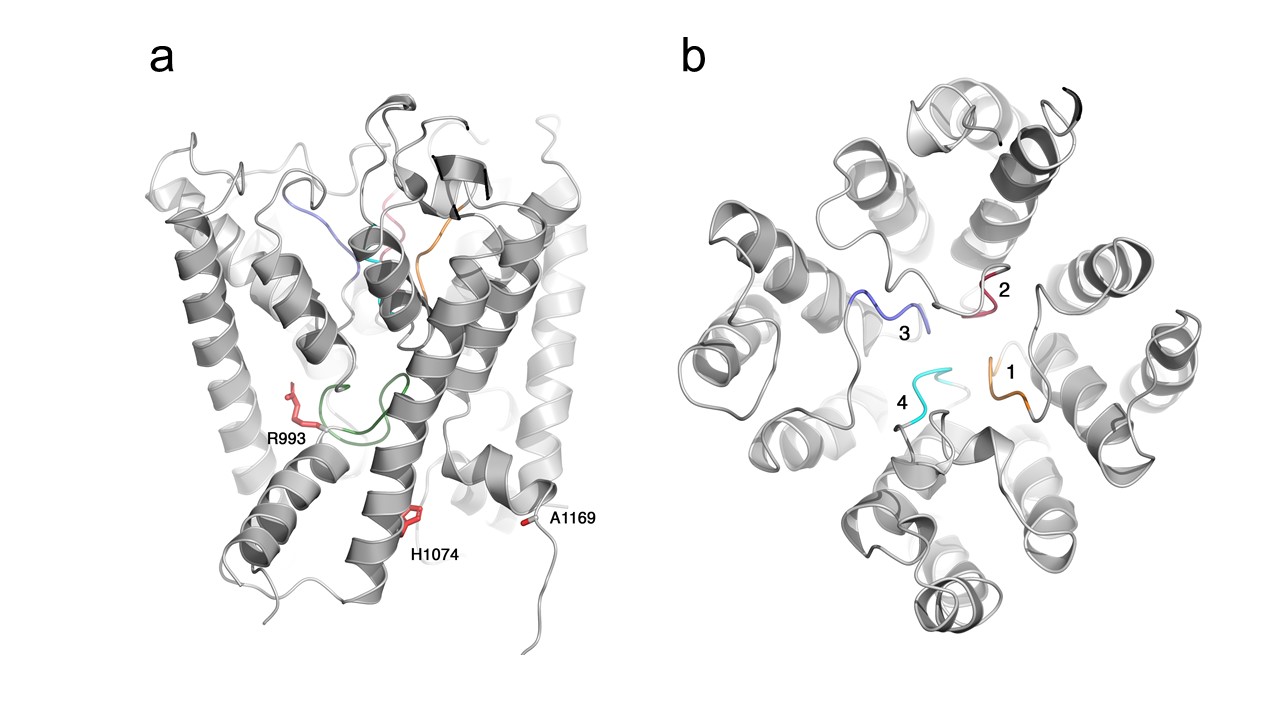
**

**Fig. S4. Cartoon showing the overall fold of the *Sc*Trk1 channel.** Side view, perpendicular to the channel (**a**)**,** and top view looking down the channel towards the selectivity filter (**b**). The four segments that constitute the selectivity filter are colored orange, red, blue and cyan, respectively. The amino acids that are subject to mutations (R993, H1074, A1169) are colored red and shown as stick objects. The intramembrane loop (IML, residues 983-992) “below” the selectivity filter is colored green.


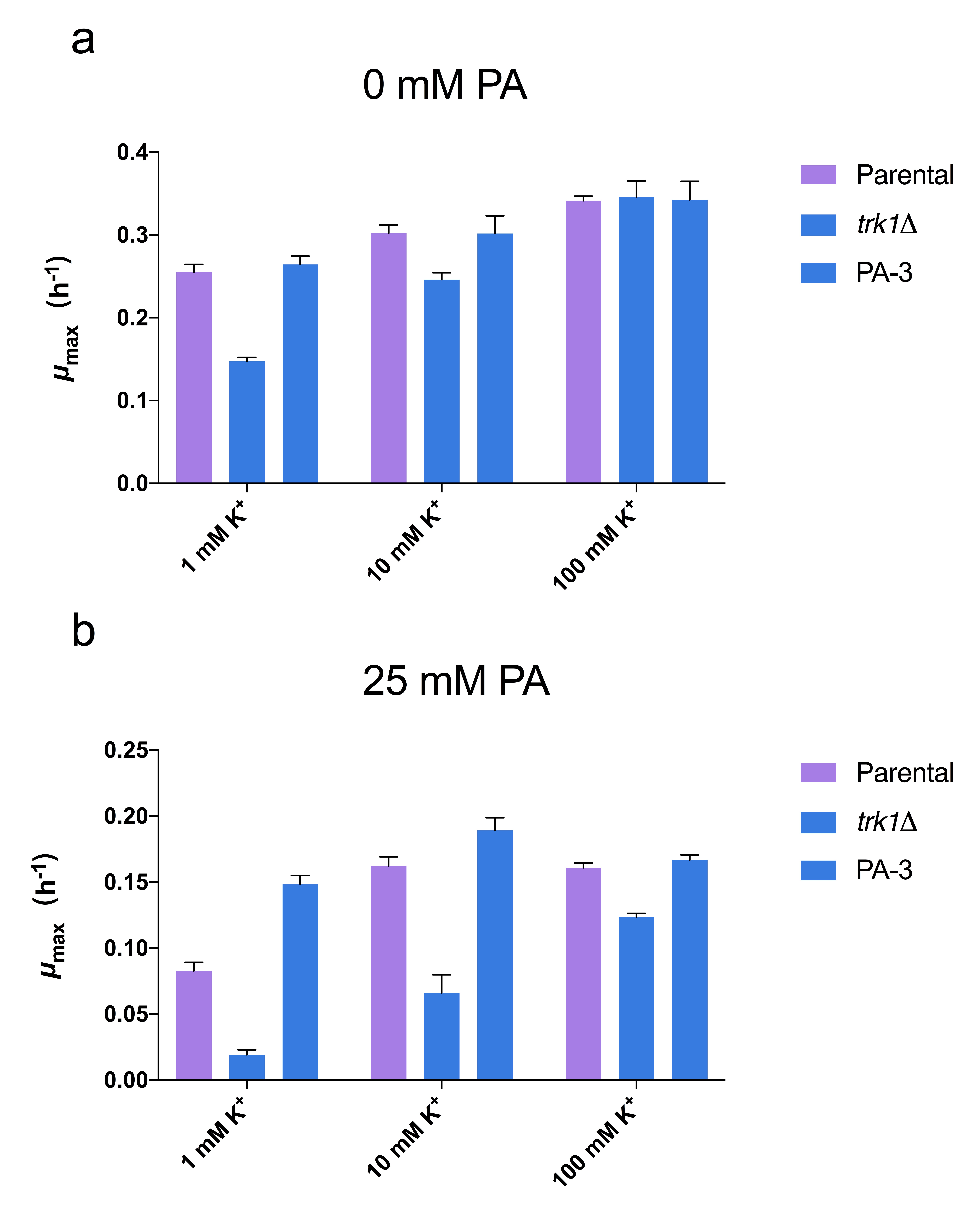


**Fig. S5. The effect of potassium concentrations and *TRK1* on the tolerance of yeast strains to PA in liquid culture.** Growth rates of WT, *trk1Δ* and the evolved isolate PA-3 were determined in minimal medium supplemented with increasing concentrations of potassium (1 mM, 10 mM, and 100 mM) with 0 mM (**a**) and 25 mM PA (**b**). Bars and error bars represent the mean and SD of triplicate cultures.

**Table S1. List of plasmids used in this study.**

| **Name** | **Details** | **Origin** |
| --- | --- | --- |
| pRS413 | Yeast centromeric plasmid, *HIS3* marker | Euroscarf |
| Cas9-gRNA-pRS423 | *pTEF1-CAS9-CYC1t-SNR52-CAN1.Y-crRNA-CYC1t -*pRS423, *HphMX* marker | This lab |
| pPGK1-CYC1t-pRS426 | *pPGK1-CYC1t*-pRS426, *URA3* and *HphMX* marker | This lab |
| pPGK1-TRK1-CYC1t-pRS426 | *pPGK1-TRK1-CYC1t*-pRS426, *URA3* and *HphMX* marker | This study |
| pPGK1-TRK1-CYC1t-pRS413 | *pPGK1-TRK1-CYC1t*-pRS413, *HphMX* and *HIS3* marker | This study |

**Table S2. List of primers used in this study.**

| **Primer name** | **5′ to 3′ sequence** |
| --- | --- |
| TRK1F | TAGATGCATCATTTGGATAATG |
| TRK1R | TTAGAGCGTTGTGCTGCT |
| TRK1-3223F | ACGGATACTGAAGACGATGGT |
| TRK1-3223R | GGTGTCCGGATAACCTAGCG |
| TRK1-3509F | TGCATTTGTGAAGGGGACAAG |
| TRK1-3509R | GCGTTGTGCTGCTCCTTTTA |
| pCRISPR-TRK1-3223 gRNAF | ATGAAAGCTGTTTCCTTAAAGTTTTAGAGCTAGAAATAGCAAG |
| pCRISPR-TRK1-3223 gRNAR | TTTAAGGAAACAGCTTTCATGATCATTTATCTTTCACTGCGGA |
| pCRISPR-TRK1-2981 gRNAF | CATTGGCCATCTCTATTCGAGTTTTAGAGCTAGAAATAGCAAG |
| pCRISPR-TRK1-2981 gRNAR | TCGAATAGAGATGGCCAATGGATCATTTATCTTTCACTGCGGA |
| pCRISPR-TRK1-3509 gRNAF | ATTGCACGATCCAGTGAGTAGTTTTAGAGCTAGAAATAGCAAG |
| pCRISPR-TRK1-3509 gRNAR | TACTCACTGGATCGTGCAATGATCATTTATCTTTCACTGCGGA |
| TRK1 seq checkF | GTTTCAATCTGTTAGCACAAG |
| TRK1 seq checkR | CATCAGAAATGTACGTAGGC |
| Hph-TRK1F | TTTTAGAAGAACGATGAGTAGAGTGCCCACATTGGCATCTCTTGAAATACGATATAAAAAATCTTTCGGCGTTTTTCGACACTGGATGGC |
| Hph-TRK1R | GTGCTGCTCCTTTTAGGATTTCGGGAATGTGTGGTCTTACGCTTAAGAGCTCCCCAACGATGTTTCACATCACGCGTTTAGCTTGCCTCG |
| Hph-upstreamF | GAGGGGATGTTATTTCAAGCAC |
| Hph-upstreamR | GCCATCCAGTGTCGAAAAACGCCGAAAGATTTTTTATATCGTATT |
| Hph-downstreamF | CGAGGCAAGCTAAACGCGTGATGTGAAACATCGTTGG |
| Hph-downstreamR | AATCGATGAGTGGGGATT |
| TRK1-Hph-knock-seqF | CTCTCGCTAAATTCCCCAATG |
| TRK1-Hph-knock-seqR | GAGGGACAATGTACTAATGGC |
| TRK1 with pRS426 overhangF | AACAAATTTAATTAAGGTTCCAAGGATGCATTTTAGAAGAACGATGAG |
| TRK1 with pRS426 overhangR | AAAGGAAAAGGCGCGCCTTGGAATTAGAGCGTTGTGCTGC |
| pRS426 with TRK1 overhangF | TAAAAGGAGCAGCACAACGCTCTAATTCCAAGGCGCGCCT |
| pRS426 with TRK1 overhangR | CTCATCGTTCTTCTAAAATGCATCCTTGGAACCTTAATTAAATTTGTT |
| TRK1-pPGK1-pRS426-checkF | GCGATCGCTCCCTCCTTCT |
| TRK1-CYC1t-pRS426-checkR | GGTTAAACGGTTCCAAGGCC |
| pRS426-pPGK1 with pRS413 overhangF | CGAGGTCGACGGTATCGATTGGGTAACGCCAGGGTTTT |
| pRS426-Hph with pRS413 overhangR | GGAATTCGATATCAAGCTTATGTGTGGAATTGTGAGCGGA |
| pRS413-with pRS426 overhangF | TCCGCTCACAATTCCACACATAAGCTTGATATCGAATTCC |
| pRS413-with pRS426 overhangR | AAAACCCTGGCGTTACCCAATCGATACCGTCGACCTCG |
| pPGK1-TRK1-CYC1t-pRS413 checkF | ATGACAGAGCAGAAAGCCCTAG |
| pPGK1-TRK1-CYC1t-pRS413 checkR | GTGTAGAAGTAGTGAACCGCGA |

**Table S3. Genotypic changes in the PA evolved populations.**

| **Gene name** | **Mutations** | | | **Gene function** |
| --- | --- | --- | --- | --- |
|  | **Lineage-1** | **Lineage-2** | **Lineage-3** |  |
| TRK1 | H1074N | R993P | A1169E | Component of the Trk1p-Trk2p potassium transport system; 180 kDa high affinity potassium transporter |
| OCT1 | / | A714V | / | Mitochondrial intermediate peptidase; may contribute to mitochondrial iron homeostasis |
| FKH2 | / | M626I | / | Forkhead family transcription factor; rate-limiting activator of replication origins |
| THP3 | / | Q469K | / | Protein that may have a role in transcription elongation; possibly involved in splicing based on pre-mRNA accumulation defect for many intron-containing genes |
| PUS9 | / | / | V404G | Mitochondrial tRNA: pseudouridine synthase |
| HNM1 | / | / | CDS176(AA59)  Truncation | Plasma membrane transporter for choline, ethanolamine, and carnitine; involved in the uptake of nitrogen mustard and the uptake of glycine betaine during hypersaline stress |

The mutations were identified from end point PA evolved populations after excluding the mutations existed in the parental strain and the strains from control conditions.
